# Death or survival from invasive pneumococcal disease in Scotland: associations with serogroups and multilocus sequence types

**DOI:** 10.1099/jmm.0.028803-0

**Published:** 2011-06

**Authors:** Donald Inverarity, Karen Lamb, Mathew Diggle, Chris Robertson, David Greenhalgh, Tim J. Mitchell, Andrew Smith, Johanna M. C. Jefferies, Stuart C. Clarke, Jim McMenamin, Giles F. S. Edwards

**Affiliations:** 1 Institute of Infection, Immunity and Inflammation, College of Medical, Veterinary and Life Sciences, University of Glasgow, 120 University Place, Glasgow G12 8QQ, UK; 2 Department of Mathematics and Statistics, University of Strathclyde, Glasgow G1 1XH, UK; 3 Scottish Haemophilus, Legionella, Meningococcal and Pneumococcal Reference Laboratory (SHLMPRL), Stobhill General Hospital, Glasgow G21 3UW, UK; 4 College of Medical, Veterinary and Life Sciences, University of Glasgow, Glasgow G12 8QQ, UK; 5 Division of Infection, Inflammation and Immunity, University of Southampton School of Medicine, Southampton SO16 6YD, UK; 6 Health Protection Agency, Southampton SO16 6YD, UK; 7 Southampton NIHR Respiratory Biomedical Research Unit, Southampton SO16 6YD, UK; 8 Health Protection Scotland, Clifton House, Clifton Place, Glasgow G3 7LN, UK

## Abstract

We describe associations between death from invasive pneumococcal disease (IPD) and particular serogroups and sequence types (STs) determined by multilocus sequence typing (MLST) using data from Scotland. All IPD episodes where blood or cerebrospinal fluid (CSF) culture isolates were referred to the Scottish Haemophilus, Legionella, Meningococcal and Pneumococcal Reference Laboratory (SHLMPRL) from January 1992 to February 2007 were matched to death certification records by the General Register Office for Scotland. This represented 5959 patients. The median number of IPD cases in Scotland each year was 292. Deaths, from any cause, within 30 days of pneumococcal culture from blood or CSF were considered to have IPD as a contributing factor. Eight hundred and thirty-three patients died within 30 days of culture of *Streptococcus pneumonia*e from blood or CSF [13.95 %; 95 % confidence interval (13.10, 14.80)]. The highest death rates were in patients over the age of 75. Serotyping data exist for all years but MLST data were only available from 2001 onward. The risk ratio of dying from infection due to particular serogroups or STs compared to dying from IPD due to all other serogroups or STs was calculated. Fisher’s exact test with Bonferroni adjustment for multiple testing was used. Age adjustment was accomplished using the Cochran–Mantel–Haenszel test and 95 % confidence intervals were reported. Serogroups 3, 11 and 16 have increased probability of causing fatal IPD in Scotland while serogroup 1 IPD has a reduced probability of causing death. None of the 20 most common STs were significantly associated with death within 30 days of pneumococcal culture, after age adjustment. We conclude that there is a stronger association between a fatal outcome and pneumococcal capsular serogroup than there is between a fatal outcome and ST.

## Introduction

Infections due to *Streptococcus pneumoniae* (the pneumococcus) remain a substantial source of morbidity and mortality in both developing and developed countries despite a century of research and the development of therapeutic interventions such as multiple classes of antibiotics and vaccination. The World Health Organization estimates that in developing countries 814 000 children under the age of five die annually from invasive pneumococcal disease (IPD) (Scott, 2007), with an estimated 1.6 million deaths affecting all ages globally (WHO, 2007).

Case fatality rates for pneumococcal meningitis cases are generally higher than non-meningitis cases (Rückinger *et al.*, 2009), and bacteraemic pneumococcal meningitis has a poorer outcome than non-bacteraemic pneumococcal meningitis (Carrol *et al.*, 2008). The mortality rate at 14 days from pneumococcal bacteraemia is generally around 17 % (Yu *et al.*, 2003) although at 30 days it has been found to be as high as 33 % in urban Scotland (Maddox & Winter, 2003). In patients with IPD, age over 65, underlying chronic disease, immunosuppression and disease severity have been significantly associated with increased mortality in multivariate analyses (Alanee *et al.*, 2007; Yu *et al.*, 2003).

Several recent studies have identified associations between pneumococcal serotypes and patient outcomes from IPD. Serotype 3 has been shown to be associated with an increased relative risk of death and serotype 1 a lower relative risk of death in Denmark (Harboe *et al.*, 2009). A recent meta-analysis identified serotypes 1, 7F and 8 as being associated with decreased relative risk of death due to pneumococcal pneumonia while serotypes 3, 6A, 6B, 9N and 19F were associated with increased relative risk of death (Weinberger *et al.*, 2010).

The primary aim of this study was to identify an association between any of the serogroups identified as causing IPD in Scotland and death at 30 days after culture of pneumococci. The secondary aim was to determine if any sequence types are significantly associated with mortality. In addition, it was of interest to assess associations between serogroups and sequence types and mortality for different age groups.

## Methods

The IPD episodes referred to in this study relate to clinical isolates (grown from blood or cerebrospinal fluid, CSF) of *S. pneumoniae* sent to the Scottish Haemophilus, Legionella, Meningococcal and Pneumococcal Reference Laboratory (SHLMPRL) from January 1992 to February 2007, identified at diagnostic microbiology laboratories in Scotland. At SHLMPRL, these isolates were grown on Columbia blood agar (Oxoid) at 37 °C under anaerobic conditions by use of an anaerobic pack (Oxoid) and after a single subculture were stored at −80 °C on Protect beads (M-Tech Diagnostics). Optochin susceptibility was confirmed by disc diffusion, and susceptibilities to penicillin, erythromycin and cefotaxime were determined using E-test strips (AB Biodisk). Breakpoints published by the British Society of Antimicrobial Chemotherapy were used to assess antimicrobial susceptibility as previously described (Cooke *et al.*, 2010). Isolates were serotyped by an established coagglutination method (Smart, 1986).

Multilocus sequence typing (MLST) was performed as described previously (Clarke & Diggle, 2002; Enright & Spratt, 1998; Jefferies *et al.*, 2003). Briefly, fragments from seven housekeeping genes, *aroE*, *gdh*, *gki*, *recP*, *spi*, *xpt* and *ddl*, were amplified from the pneumococcal lysate with the primers described by Enright & Spratt (1998) by using a single PCR. The amplified DNA was cleaned as previously described (Clarke & Diggle, 2002; Sullivan *et al.*, 2006). The cleaned amplified DNA was then sequenced with the same primer set using the DYEnamic ET Terminator sequencing kit (Amersham Biosciences). These procedures were carried out on a liquid handling robotic platform (MWG-Biotech) and a MegaBACE 1000 DNA sequencer (Amersham Biosciences). Analysis of the sequence data and subsequent assignment of a sequence type (ST) was performed as described previously (Diggle & Clarke, 2002).

The database of these isolates, which are stored at SHLMPRL, was matched to death certification records (Kendrick & Clarke, 1993) by the General Register Office for Scotland. Only the first isolate received was used to indicate an episode of IPD in cases when multiple isolates were received from the same patient. Ethical approval for data matching was received from North Glasgow University Hospitals Glasgow Royal Infirmary Research Ethics Committee (REC reference 07/S0704/27). Deaths, from any cause, within 30 days of pneumococcal culture from blood or CSF were considered to have IPD as a contributing factor.

To determine associations between mortality and serogroup or ST, the risk ratio of dying within 30 days from IPD due to particular serogroups or STs compared to dying within 30 days from IPD attributable to all other serogroups or STs was calculated. The risk ratio (RR) is calculated as (*a*/(*a+b*))/(*c*/(*c+d*)), where *a* is the number of deaths within 30 days of developing IPD attributable to the serogroup or ST under scrutiny (serogroup *i* or ST *i*), *b* is the number who survived more than 30 days of serogroup *i* or ST *i* IPD, *c* is the number of deaths within 30 days attributable to all other serogroups or STs (i.e. all non-serogroup *i* or ST *i* IPD deaths), and *d* is the number of survivors of more than 30 days of disease from all other serogroups or STs. Each serogroup or ST found in IPD was compared to IPD cases from all other serogroups or STs so that an estimate of the risk ratio could be obtained for each of the serogroups or STs relative to all others, rather than compared to a single baseline serogroup or ST. This approach of floating absolute risk (Easton *et al.*, 1991) was employed in a study establishing the invasive disease potential of serotypes and STs among children in Oxford, England (Brueggemann *et al.*, 2003). A risk ratio of 1 indicates that an individual with serogroup *i* or ST *i* is as likely to die within 30 days of IPD as an individual with IPD from a serogroup or ST other than *i*. A risk ratio of greater than 1 can be interpreted as being indicative of an increased probability of death within 30 days of IPD due to serogroup *i* or ST *i* invasive disease, whilst a risk ratio of less than 1 is indicative of a reduced probability for the serogroup or ST to cause death within 30 days.

To investigate if certain serogroups or STs are associated with a greater or reduced risk of death within 30 days of IPD, Fisher’s exact test was used. This test is more appropriate than the χ^2^ test of association as many serogroups and STs are rarely observed in IPD and some more common serogroups may be rarely observed in IPD cases with fatal outcomes. The Cochran–Mantel–Haenszel test was used to carry out adjustment for age when testing the association between serogroups or STs and mortality. The null hypothesis of this test is no association between the two categorical variables across all strata; the alternative hypothesis is that there is an association between the two variables in at least one of the strata. The Bonferroni correction factor was used to adjust for multiple testing. Logistic regression was used to determine whether or not the proportion of fatalities remained constant over time.

Analyses were performed using R version 2.8.0 (R Development Core Team, 2008).

## Results

During the study period 833 of the 5959 patients with IPD [13.95 %; 95 % CI (13.10, 14.80)] died within 30 days of the sample submission to SHLMPRL. There were 7 (0.12 %) patients that could not be matched to the data on death certification records from the General Register Office for Scotland.

Examination of the number of cases of IPD for each of the years between 1992 and 2007 showed an increase from a minimum of 29 cases of IPD observed in 1992 to a maximum of 697 cases in 2006. The number of IPD cases appeared relatively low initially and generally increased each year, until 2000. The apparent increased number of cases of IPD may be due to improved surveillance rather than a real increase in the number of cases of IPD in Scotland during that period. On average, there were approximately 372 cases (median 292 cases) of IPD in Scotland each year.

**Fig. 1.**
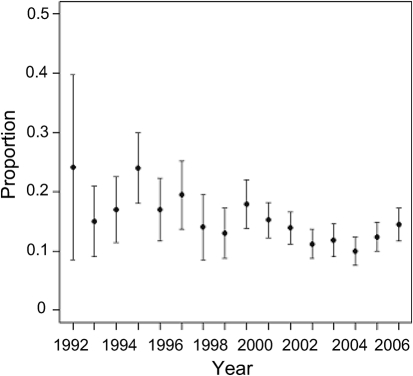
Plot of the proportion of IPD fatalities each year in Scotland from 1992 to 2007 (95 % confidence intervals shown). A logistic regression analysis showed that the proportions of fatalities were not constant over time (*P*<0.0001) and that the risk of dying within 30 days reduced by 3.92 % each year [95 % CI (2.48 %, 5.99 %)]. After adjustment for age group and gender, the results showed that the risk of dying within 30 days reduced on average by 5.08 % each year [95 % (CI 3.19 %, 6.94 %)].

The proportion of fatalities attributable to IPD did not increase over the period of study even though the number of identified cases increased (Fig. 1). Case fatality decreased from 24 % (7 of 29 cases) in 1992 to 12 % (86 of 697 cases) in 2006. The improved surveillance of IPD in Scotland may have contributed to this as over time it is likely that isolates have been referred from more cases associated with less severe disease than would have been the case in the 1990s, when isolates from severe or complicated IPD were mainly referred. Other influences such as improved case management cannot be excluded, however.

**Fig. 2.**
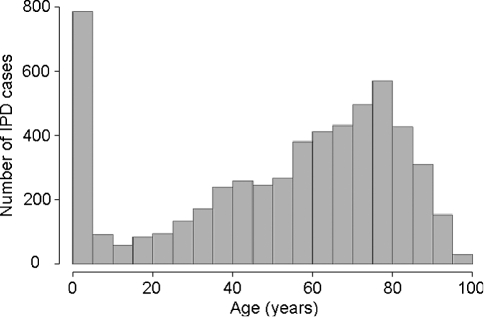
Histogram of the age distribution of individuals with IPD in Scotland from 1992 to 2007.

**Fig. 3.**
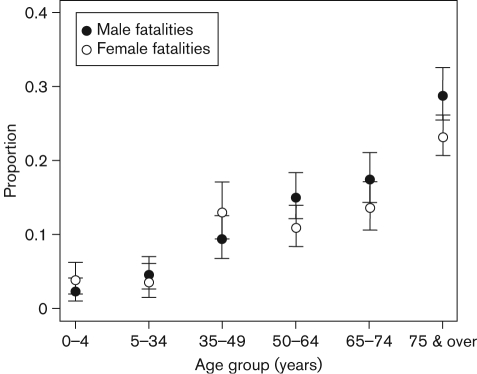
Plot of the proportion of fatalities from IPD each year in Scotland within gender by age group from 1992 to 2007 (95 % confidence intervals shown).

Fig. 2 shows the distribution of age for the patients with IPD, with age ranging from 0 to 99 years. IPD appears most common for those under 10 years of age and those between 70 and 80 years of age. Information regarding age was missing from 301 patients. Age was categorized into six strata: 0–4 years, 5–34 years, 35–49 years, 50–64 years, 65–74 years, and 75 years and over. Of the 5959 patients, 3015 (50.60 %) were male and 2913 (48.88 %) were female; 31 patients had missing gender information. Fig. 3 shows the proportion of fatalities of IPD within gender by age group. This figure shows that a marginally higher proportion of females who acquired IPD had fatal outcomes than the proportion of males who acquired IPD within the age groups 0–4 years and 35–49 years. For all other age groups, the opposite is true. Fig. 3 also shows that the highest proportion of fatalities occurs in the 75 years and over age group for both genders and the lowest proportion of fatalities is observed in the 0–4 year old age group.

### Serogroup analysis

Thirty-five different serogroups were observed causing IPD in Scotland between January 1992 and December 2007. Serogroup 3 had the highest proportion of deaths from IPD within 30 days with 24 % (85 of 349 cases, RR 1.99, *P*<0.001) resulting in death. Serogroups 19 and 23 had the next-highest fatality rates, at 18 % (83 of 453 cases, RR 1.39, *P* = 0.01) and 15 % (59 of 385 cases, RR 1.12, *P* = 0.40) respectively. Serogroup 1 had the lowest rate of fatality within 30 days, with only 5 % (26 of 513 cases, RR 0.33, *P*<0.001) of cases resulting in death. Serogroup 7 also has a relatively low percentage of IPD fatality, at only 8 % (23 of 272 cases, RR 0.57, *P* = 0.01).

Table 1 shows the risk ratio and Bonferroni-adjusted 95 % confidence interval for the risk of death from each of the 20 most common serogroups identified as causing IPD in Scotland for both Fisher’s exact test and the age-stratified Cochran–Mantel–Haenszel test.

**Table 1.** Results from Fisher’s exact test and age-stratified Cochran–Mantel–Haenszel test of association between mortality and the 20 most common serogroups A *P*-value of less than 0.0025 is required for significance.

Serogroup	Died	Total	Risk ratio	Bonferroni-adjusted 95 % CI	*P*-value	Bonferroni-adjusted 95 % CI (stratified by age group)	*P-*value (stratified by age group)
14	106	919	0.81	(0.60, 1.08)	0.02	(0.62, 1.10)	0.05
9	79	537	1.07	(0.75, 1.51)	0.60	(0.73, 1.40)	0.98
1	26	513	0.33	(0.18, 0.60)	<1×10^−5^	(0.17, 0.60)	<1×10^−4^
6	62	463	0.96	(0.64, 1.42)	0.78	(0.67, 1.39)	0.82
19	83	453	1.39	(0.98, 1.96)	0.01	(1.05, 1.94)	<1×10^−3^
4	57	410	1.00	(0.66, 1.51)	1.00	(0.65, 1.42)	0.77
23	59	385	1.12	(0.74, 1.69)	0.40	(0.68, 1.43)	0.99
8	41	357	0.80	(0.49, 1.31)	0.18	(0.49, 1.21)	0.07
3	85	349	1.99	(1.39, 2.85)	<1×10^−5^	(1.12, 2.02)	<1×10^−4^
7	23	272	0.57	(0.30, 1.09)	0.01	(0.41, 1.29)	0.07
18	17	213	0.54	(0.25, 1.14)	0.01	(0.37, 1.45)	0.16
12	30	186	1.19	(0.66, 2.15)	0.39	(0.73, 2.04)	0.34
22	26	170	1.12	(0.59, 2.1)	0.57	(0.56, 1.68)	0.96
11	27	106	2.11	(1.09, 4.1)	<2×10^−3^	(1.06, 2.79)	<0.01
20	9	106	0.57	(0.2, 1.63)	0.12	(0.22, 1.55)	0.03
33	11	76	1.05	(0.39, 2.78)	0.87	(0.48, 2.20)	0.80
15	12	72	1.24	(0.48, 3.19)	0.49	(0.6, 2.62)	0.68
10	7	44	1.17	(0.34, 4.04)	0.66	(0.46, 3.37)	0.79
16	13	34	3.82	(1.32, 11.05)	<1×10^−3^	(1.35, 4.57)	<2×10^−3^
31	10	34	2.57	(0.83, 7.99)	0.02	(1.11, 4.66)	0.02

There is significant evidence of an association between mortality and serogroups 1, 3 and 16. Significant evidence of an association between serogroup 11 and mortality was found using Fisher’s exact test but not the Cochran–Mantel–Haenszel test and serogroup 19 was found to be associated with mortality using the Cochran–Mantel–Haenszel test but not Fisher’s exact test. Serogroup 3 has a risk ratio of dying of 1.99 relative to all other serogroups; serogroup 11 has a risk ratio of 2.11, serogroup 16 has the largest risk ratio of 3.82 and serogroup 19 has a risk ratio of 1.39. Therefore, there is an increased risk of a fatal outcome within 30 days of a diagnosis of IPD attributable to any of those serogroups rather than any other serogroup IPD. Serogroup 1 has a risk ratio of 0.33. Therefore, there is a reduced risk of fatality within 30 days of a diagnosis of IPD attributable to this serogroup compared to all other serogroups.

Serotype information was available from 2003 in Scotland. IPD from serogroups 6, 7 and 19 was assessed from 2003 to 2007 to investigate associations between the serotypes from within these serogroups and fatality within 30 days of a diagnosis of IPD. No significant associations were found between any of the serotypes and mortality.

### MLST analysis

There were 371 different STs identified in cases of IPD between 2001 and 2007. Of the 20 most common disease-causing STs, ST180 has the worst outcome, with 22 % of cases (38 of 174 cases, RR 1.98, *P*<0.001) ending in death at 30 days post-diagnosis. ST306 has the best outcomes, with only approximately 3 % of cases (8 of 250 cases, RR 0.23, *P*<0.001) ending in death.

Table 2 displays the risk ratio and Bonferroni-adjusted 95 % confidence interval for the risk of death of each of the 20 most common STs identified in IPD in Scotland. There is significant evidence of an association between fatal outcomes and ST306, ST180 and ST191 from Fisher’s Exact Test but not from the Cochran–Mantel–Haenszel test. For ST306 and ST191 there is a reduced risk of death. For ST180 there is an increased risk of death. No significant associations were found using the Cochran–Mantel–Haenszel test to adjust for the different age strata. Tables 3 and 4 show that the STs that are significantly associated with fatal outcomes are predominantly associated with just one serogroup. Apart from serogroups 1 and 19 (where there are several STs represented in each of these serogroups) there is often a dominant ST for the significant serogroups.

**Table 2.** Results from Fisher’s exact test and age-stratified Cochran–Mantel–Haenszel test of association between mortality and the 20 most common STs A *P*-value of less than 0.0025 is required for significance.

ST	Died	Total	Risk ratio	Bonferroni-adjusted 95 % CI	*P*-value	Bonferroni-adjusted 95 % CI (stratified by age group)	*P*-value (stratified by age group)
9	34	360	0.74	(0.44, 1.25)	0.08	(0.48, 1.30)	0.17
306	8	250	0.23	(0.08, 0.69)	<1×10^−6^	(0.18, 1.18)	<0.01
162	30	241	1.01	(0.57, 1.78)	0.92	(0.58, 1.63)	0.90
53	17	199	0.66	(0.31, 1.40)	0.10	(0.32, 1.29)	0.04
180	38	174	1.98	(1.16, 3.38)	<1×10^−4^	(0.96, 2.33)	0.01
191	8	171	0.35	(0.12, 1.03)	<8×10^−4^	(0.21, 1.36)	0.02
124	16	140	0.91	(0.42, 2.01)	0.90	(0.44, 1.70)	0.41
199	19	121	1.32	(0.63, 2.77)	0.26	(0.74, 2.51)	0.27
246	16	111	1.19	(0.53, 2.66)	0.47	(0.63, 2.51)	0.48
218	14	111	1.02	(0.44, 2.40)	0.88	(0.54, 2.28)	0.88
311	15	99	1.27	(0.55, 2.91)	0.36	(0.58, 2.31)	0.81
227	6	97	0.47	(0.13, 1.66)	0.06	(0.28, 2.45)	0.50
433	10	80	1.01	(0.37, 2.78)	1.00	(0.39, 2.13)	0.64
205	7	68	0.81	(0.25, 2.69)	0.71	(0.38, 2.68)	0.88
176	11	64	1.47	(0.55, 3.96)	0.25	(0.80, 3.44)	0.20
206	8	63	1.03	(0.33, 3.20)	0.85	(0.49, 2.89)	0.92
113	6	60	0.79	(0.22, 2.87)	0.70	(0.39, 3.20)	0.96
62	9	53	1.45	(0.48, 4.34)	0.29	(0.63, 3.22)	0.61
36	9	52	1.48	(0.49, 4.45)	0.29	(0.52, 2.88)	0.88
235	7	49	1.18	(0.35, 4.02)	0.66	(0.43, 3.10)	0.97

**Table 3.** STs associated with the serogroups significantly associated with 30 day mortality

Serogroup	ST (number of isolates)
1	306 (243), 227 (96), 1310 (3), 1809 (3), 9 (2), 1882 (2), 2126 (2), 53 (1), 138 (1), 162 (1), 176 (1), 179 (1), 180 (1), 199 (1), 205 (1), 228 (1), 246 (1), 304 (1), 312 (1), 618 (1), 1239 (1), 1247 (1), 1311 (1), 1346 (1), 1597 (1), 2126 (1), 2135 (1), 3229 (1), 3230 (1)
3	180 (172), 260 (4), 232 (4), 1003 (3), 1468 (3), 1220 (3), 233 (3), 53 (2), missing (2), 1253 (2), 2263 (2), 458 (2), 162 (2), 312 (1), 378 (1), 862 (1), 1300 (1), 1344 (1), 1377 (1), 1682 (1), 1765 (1), 1867 (1), 1887 (1), 2119 (1), 2979 (1)
11	62 (53), 408 (6), 513 (3), 199 (1), 446 (1), 1180 (1), 1219 (1), 1304 (1)
16	570 (6), 30 (3), 414 (3), 863 (1), 1382 (1), 2127 (1), 2130 (1), 2262 (1), 2827 (1)
19	199 (88), 162 (59), 426 (14), 309 (7), 416 (6), 177 (5), 179 (4), 688 (4), 1201 (4), 9 (3), 43 (3), 246 (3), 420 (3), 422 (3), 667 (3), 1718 (3), 191 (2), 193 (2), 236 (2), 276 (2), 419 (2), 423 (2), 424 (2), 645 (2), 654 (2), 686 (2), 1002 (2), 1218 (2), 1359 (2), 53 (1), 58 (1), 66 (1), 81 (1), 124 (1), 156 (1), 165 (1), 176 (1), 251 (1), 271 (1), 306 (1), 312 (1), 395 (1), 425 (1), 438 (1), 450 (1), 459 (1), 462 (1), 476 (1), 482 (1), 494 (1), 644 (1), 655 (1), 697 (1), 799 (1), 826 (1), 839 (1), 994 (1), 1035 (1), 1233 (1), 1258 (1), 1298 (1), 1545 (1), 1757 (1), 2067 (1), 2076 (1), 2220 (1), 2265 (1), 2365 (1), 2370 (1), 3211 (1), 3217 (1)

**Table 4.** Serogroups associated with the STs significantly associated with 30 day mortality

ST	Serogroup (number of isolates)
306	1 (243), missing (2), 4 (1), 6 (1), 14 (1), 18 (1), 19 (1)
180	3 (172), missing (1), 6 (1), 33 (1)
191	7 (164), 6 (2), 19 (2), 4 (1), 5 (1), 14 (1)
227	1 (96), 6 (1), missing (1)

Considering the serogroups significantly associated with 30 day mortality, within the cases of serogroup 1 there is no evidence that death from IPD is associated with ST (*P* = 0.12), and compared to ST306, ST227 has a risk ratio of 1.64 [(95 % (CI 0.58, 4.63)], as illustrated in Table 5. Similarly, within the cases of serogroup 19 there is no evidence that fatal outcome is associated with ST (*P* = 0.97). Compared to ST199, ST162 has a risk ratio of 1.18 [95 % CI (0.58, 2.40)] and ST426 has a risk ratio of 0.99 [95 % CI (0.26, 3.73)].

**Table 5.** Risk ratio for fatal outcome for the STs linked to the serogroups significantly associated with death at 30 days after diagnosis of IPD For serogroup 1, MLST data were not known for 140 cases of IPD; for serogroup 3, MLST data were not known for 134 cases of IPD; for serogroup 11, MLST data were not known for 39 cases of IPD and for serogroup 19, MLST data were not known for 173 cases of IPD; therefore ‘All non-missing’ indicates all cases of (serogroup 1, serogroup 3, serogroup 11 or serogroup 19) IPD with complete MLST data available for analysis. Inf, infinity; RR, risk ratio; LCL, lower confidence limit; UCL, upper confidence limit.

	Number	Number died	Percentage	RR	LCL	UCL	*P*-value	Overall *P*-value
**Serogroup 1**								
All cases	513	26	5.1					
All non-missing	373	13	3.5					
ST306	243	7	2.9	1.00	–	–	–	
ST227	96	6	6.3	1.64	0.58	4.63	0.33	
Other STs	34	0	0.0	0.00	0.00	Inf	0.99	0.12
**Serogroup 3**								
All cases	349	85	24.4					
All non-missing	215	53	24.7					
ST180	172	38	22.1	1.00	–	–	–	
Other STs	44	14	31.8	1.39	0.84	2.29	0.22	0.23
**Serogroup 11**								
All cases	106	27	25.5					
All non-missing	67	13	19.4					
ST62	53	9	17.0	1.00	–	–	–	
Other STs	14	4	28.6	1.00	0.35	2.83	0.42	0.43
**Serogroup 19**								
All cases	453	83	18.3					
All non-missing	280	45	16.1					
ST199	88	14	15.9	1.00	–	–	–	
ST162	59	10	16.9	1.18	0.58	2.40	0.68	
ST426	14	2	14.3	0.99	0.26	3.73	0.96	
Other STs	119	19	16.0	1.00	0.54	1.88	0.96	0.97

Furthermore, no evidence was found of an association between death from IPD and ST for either serogroup 3 (*P* = 0.23) or serogroup 11 (*P* = 0.43) comparing IPD from the dominant ST (ST180 and ST570, respectively) to all other STs linked to each of these serogroups. There were too few cases of serogroup 16 IPD to consider associations between ST and IPD for this serogroup.

## Discussion

Early observations in the history of pneumococcal research suggested that particular serotypes had a propensity for more severe disease manifestations and that some serotypes were more commonly associated with a fatal outcome. In the pre-antibiotic era, serotype 3 pneumococcal pneumonia was associated with high case fatality rates and serotype 1 with lower case fatality rates when no treatment other than symptomatic relief was administered (Avery *et al.*, 1917; Cowan *et al.*, 1932). The introduction of penicillin had less effect on case fatality rates from serotype 3-associated pneumococcal pneumonia than case fatality rates due to other serotypes (Austrian & Gold, 1964; Macfarlane *et al.*, 1982).

In Sweden, greater disease severity has been associated with serotypes 3, 6A, 6B, 19A and 19F while in the same study, serotypes 1, 4 and 7F had the least severe disease (Sjöström *et al.*, 2006). Serotypes 3, 6A and 19F also had high case fatality rates in this study (Sjöström *et al.*, 2006) while serotype 19A alone had a high case fatality rate in another Swedish study (Berg *et al.*, 2006). Serotypes 1 and 7F had low case fatality rates in both Swedish studies (Berg *et al.*, 2006; Sjöström *et al.*, 2006). Recently in Germany (Rückinger *et al.*, 2009), a study of 494 children identified an overall case fatality rate of 5.3 %. Serotype 7F had the highest case fatality rate (14.8 %) followed by serotype 23F (8.3 %) and serotype 3 (8.3 %). In the Netherlands, serotypes 3, 19F, 23A, 16F, 6B, 9N and 18C were also recently associated with increased case fatality rates (Jansen *et al.*, 2009). The largest study assessing serotype association with death from 18 858 patients with IPD was published from Denmark and found (in patients over age 5 years) that serotypes 31, 11A, 35F, 17F, 3, 16F, 19F, 15B and 10A were associated with higher mortality when compared to serotype 1, but no associations with ST were made (Harboe *et al.*, 2009). The 30 day mortality overall was 18 % and in children under 5 years old it was 3 % (Harboe *et al.*, 2009).

A prospective multi-centre study of 796 consecutive patients from 10 countries (South Africa, USA, Sweden, Spain, New Zealand, Taiwan, Argentina, Brazil, Hong Kong and France) examined clinical outcome and mortality at 14 days after the first positive blood culture for *S. pneumoniae* (Alanee *et al.*, 2007) and assessed for associations with particular serotypes categorized as invasive (serotypes 1, 5 and 7), paediatric (serotypes 6, 9, 14, 19 and 23) and conjugate vaccine associated (serotypes 4, 6B, 9V, 14, 18C, 19F and 23F). This study focused predominantly on adults, did not include patients from the UK and did not look for associations between outcome and STs of pneumococci. In fact, although it is recognized that invasive capacity of the pneumococcus is dependent on both serotype and genomic content (Garau & Calbo, 2007) there is little published work which investigates whether there is an association between pneumococcal ST or clonal complex and disease outcome. Sjöström *et al.* (2006) performed MLST on 105 pneumococcal isolates and related these to disease severity by APACHE II score and case fatality rate. Although only assessing between 3 and 41 isolates of individual STs, they did identify ST180 as having a high case fatality rate (Sjöström *et al.*, 2006).

We chose death or survival at 30 days after culture of pneumococci from blood or CSF as our end point although other similar studies have used 14 days (Alanee *et al.*, 2007; Yu *et al.*, 2003). Up to 43 % of deaths from pneumococcal disease have been noted to occur in the first 24 h of hospital admission (Austrian & Gold, 1964) and up to 64 % of deaths occur within 5 days of hospital admission (Ortqvist *et al.*, 1993). As we did not access death certificate records or patient notes for the primary cause of death we cannot be certain that every death attributed to IPD was directly caused by IPD but such a significant event within 30 days of death is likely to have been contributing to the fatal outcome in the majority of cases.

It would be advantageous to perform this entire analysis using serotypes rather than serogroups. However, during the early years of the SHLMPRL strain collection and database, investigation of pneumococcal serotyping using factors for subtypes was not performed and so to be able to utilize all the data available to us we performed this analysis predominantly on serogroups. As serotypes 1 and 3 are equivalent to serogroup 1 and 3 (as they have no subtypes) this does not influence our findings for these serogroups. In the years since 2003, serotyping data are complete and so the data have been used to investigate whether associations can be identified with fatal outcome for individual serotypes, such as serotype 7F, which have previously been associated with high case fatality rates (Rückinger *et al.*, 2009).

Pneumococcal conjugate vaccination with a 7-valent vaccine which protects against serotypes 4, 6B, 9V, 14, 18C, 19F and 23F (Prevnar, Wyeth) was introduced in Scotland in September 2006 for infants. It is unlikely that this will have influenced our results substantially as mortality rates in the under 2 years age group (who would be directly affected by the introduction of conjugate vaccination) were low prior to vaccine introduction and the serotypes covered are not associated with greater risk of death in our analysis. From winter 2003, the 23-valent polysaccharide vaccine (Pneumovax II, Aventis Pasteur) was recommended to those aged 65 years and older in Scotland. Analysis of the impact of the first year of this intervention estimated 65 % uptake of vaccine but did not identify any significant impact on mortality due to IPD (Mooney *et al.*, 2008).

In this analysis, we were unable to look for associations between particular serogroups or STs and social deprivation scores or patient co-morbidities as such data are not submitted to SHLMPRL nor can they be extrapolated from other databases using the rudimentary epidemiological information which is collected. Such associations are highlighted in the study by Alanee *et al.* (2007), where age over 65 years, underlying chronic disease, immunosuppression and severity of illness were identified as independent risk factors significantly associated with disease mortality. Even so, in Scotland, effects of social deprivation on the incidence of IPD and the effect of underlying medical conditions on case fatality rates and the incidence of IPD have been previously documented (Kyaw *et al.*, 2003). Cases which featured in that analysis also feature in this analysis of outcome although the effects of variables other than age cannot be accounted for in our analysis. Chen *et al.* (2009) in Taiwan also assessed the role of comorbidities on outcome through multivariate analysis in their study of children with IPD (which included complicated pneumonia and meningitis) and found that penicillin resistance (MIC≥2 μg ml^−1^) was associated with mortality as an independent risk factor. Penicillin resistance in pneumococci was not shown to be associated with death in the analysis by Yu *et al.* (2003), which assessed clinical outcome of hospitalized adults with blood cultures which were positive with growth of *S. pneumoniae*. Between 1999 and 2007 only 7 of 4727 pneumococcal isolates causing IPD in Scotland were fully penicillin resistant (Cooke *et al.*, 2010). Although it is now recognized that penicillin breakpoints should be set lower in cases of meningitis than in non-meningitis cases (for instance the European Committee on Antimicrobial Susceptibility Testing advise reporting of penicillin resistance in cases of meningitis if the MIC is >0.064 µg ml^−1^), our previous analysis identified only 6 of 171 CSF isolates with MICs between 0.12 and 1 µg ml^−1^ and none with an MIC≥2 µg ml^−1^ (Cooke *et al.*, 2010); therefore we do not consider penicillin resistance to be substantially influencing mortality from pneumococcal meningitis in the Scottish population, as the vast majority of CSF isolates are fully susceptible to penicillin. In bacteraemic pneumococcal pneumonia, some evidence suggests improved outcome when treatment involves use of a macrolide (Martínez *et al.*, 2003; Metersky *et al.*, 2007; Weiss *et al.*, 2004) or combination antibiotic therapy (Baddour *et al.*, 2004; Waterer *et al.*, 2001) although other investigators have found no association between initial antibiotic choice and outcome (Aspa *et al.*, 2006).

Unfortunately the retrospective nature of this study and the lack of access to patients’ clinical notes and data regarding antibiotic prescribing mean that it is impossible for us to account for confounding by recognized independent risk factors for mortality. Even so, it is also worth reiterating the observation of Garau & Calbo (2007) that although the above-noted independent risk factors are important in determining outcome of IPD, there are substantial numbers of patients with IPD who fit a category of young adult with no pre-existing comorbidities, ‘in whom the infecting serotype becomes the determinant factor of outcome’.

It is of note that ST306 had a significant *P*-value before accounting for age with the Cochran–Mantel–Haenszel test analysis. ST306 [predominantly the Pneumococcal Molecular Epidemiology Network (PMEN) clone Sweden^1^ ST306] has now become the commonest ST in Scotland (Jefferies *et al.*, 2010; Cooke *et al.*, 2010; Lamb *et al.*, 2008). As further cases occur due to ST306 it may be that the *P*-value for this ST reaches significance after accounting for age. The possibility exists that ST306 may be associated with reduced probability of death but that at present we are unable to clearly demonstrate such an association. ST306 is now also the dominant ST to be associated with serotype 1 in Scotland (Jefferies *et al.*, 2010; Lamb *et al.*, 2008). It is interesting that no deaths in children due to serotype 1 were identified in studies from Germany (Rückinger *et al.*, 2009), in keeping with the hypothesis that serotype 1 is associated with milder disease (Sjöström *et al.*, 2006). It is known that different pneumococcal serotypes produce different inflammatory responses in animal models, influencing disease outcome (Engelhard *et al.*, 1997; Mizrachi-Nebenzahl *et al.*, 2003). Interestingly, ST306 and ST191 have been found to induce a low tumour necrosis factor (TNF) response and be effectively cleared from the bloodstream of infected mice (Sandgren *et al.*, 2005). ST306 has been found not to cause lethal murine disease (Sandgren *et al.*, 2005). Our results would be consistent with this also being possible in a human population. The association that ST191 may have a reduced risk of death is consistent with the findings of Sjöström *et al.* (2006), who on assessing 34 ST191 isolates found a case fatality rate of zero for this ST.

Although serotype 3 is associated with an increased relative risk of death from IPD in Danish adults (Harboe *et al.*, 2009; Martens *et al.*, 2004), in children, serotype 3-associated ST180 pneumococci have been identified as having an odds of invasiveness which was significantly associated with asymptomatic carriage (Brueggemann *et al.*, 2003). Recently in Germany, serotype 3-associated IPD has been shown to also have one of the highest case fatality rates in children (Rückinger *et al.*, 2009). The seemingly contradictory finding that serotype 3 pneumococci can cause disease with a high associated mortality in some individuals while being harmlessly carried in the nasopharynx of others has been recognized since the early 20th century (Blake, 1931). An association between serotype 3 pneumococci causing invasive and severe disease more commonly in the elderly than in children is also an established observation (Blake, 1931; Cecil *et al.*, 1927) which remains true in several countries (Guimbao Bescós *et al.*, 2003; Inostroza *et al.*, 2001; Kyaw *et al.*, 2003; Martin & Brett, 1996; Rahav *et al.*, 1997; Shapiro & Austrian, 1994). It is therefore not surprising that serotype 3 has been shown in this analysis to be significantly associated with a fatal outcome even when age is taken into account. It is interesting that ST180 was associated with fatal outcomes (Fisher’s exact test) before accounting for age (Cochran–Mantel–Haenszel test). Sjöström *et al.* (2006) also identified ST180 as having a high case fatality rate. This may be a consequence of a strong association between ST180 and serotype 3. ST180 has been associated with a serotype 19F capsule in Germany and non-typable isolates in South Korea but globally is predominantly associated with the serotype 3 capsule.

### Conclusions

We conclude from this analysis that there is a stronger association between a fatal outcome and pneumococcal capsular serogroup than there is between fatal outcome and multilocus ST. As the over-75 age group has the highest death rates associated with IPD and the implicated serogroups are included in the 23-valent polysaccharide vaccine (Pneumovax II, Sanofi Pasteur) which is offered to all aged over 65 years in Scotland, there may be benefit in further promotion of pneumococcal vaccination in the over 65 age group, although there is debate over the effectiveness of this vaccine (Huss *et al.*, 2009). These results also have an application in determining future pneumococcal vaccine formulations.

More recent higher-valency conjugate vaccine formulations which include serotype 3 may have an effect in reducing deaths from IPD in infants (and possibly in adults through herd immunity) while the introduction of serotype 1 into conjugate vaccine formulations may reduce morbidity from IPD but may have less effect on mortality from IPD.
